# Hologenomics of xylotrophic bivalves reveals a minimalist, remote-acting evolutionary strategy of wood digestion

**DOI:** 10.64898/2026.07.25.740686

**Published:** 2026-07-28

**Authors:** Hao Song, Biyang Xu, Yang Guo, Cong Zhou, Meijie Yang, Yantao Liu, Zhaoshan Zhong, Chun-Yang Li, Xiaolin Tian, Yanyan Wang, Ron Flatau, Minxiao Wang, Tao Zhang, Daniel L. Distel, Yuanning Li

## Abstract

Wood constitutes the largest reservoir of biogenic carbon on Earth, yet remarkably few animals can exploit it. While terrestrial wood-feeders like termites rely on highly diverse gut microbiomes, xylotrophic marine bivalves have evolved a fundamentally different approach: a spatially segregated system where intracellular gill symbionts produce enzymes that act remotely within a nearly sterile cecum. However, the genetic and evolutionary basis of this unique symbiosis remains largely elusive. Here, we integrate hologenomics, transcriptomics, and biochemistry of a shallow-water shipworm ( *Teredo navalis* ) and a deep-sea borer ( *Xyloredo* sp.). We find that despite diverging approximately 147 million years ago and occupying drastically different habitats, these bivalves maintain a strictly conserved ancestral karyotype and a shared genomic architecture for wood digestion. Our models reveal a clear host-symbiont division of labor. The host genome is specialized for lignin modification and targeted enzyme transport, whereas a highly streamlined symbiont community is responsible for core polysaccharide degradation. Central to this minimalist strategy is a lineage-specific GH5–GH6 dual-catalytic enzyme. By sharing amino acids across proximal binding pockets, this fusion protein unites endo- and exo-cellulase activities, enabling highly synergistic cellulose cleavage without the need for complex microbial communities. Ultimately, our comparative analysis with terrestrial models demonstrates that these marine invertebrates achieve efficient biomass degradation not through microbial expansion, but through extreme functional streamlining and molecular innovation, offering a distinct evolutionary paradigm for marine carbon cycling.

## Full Text Availability

The license terms selected by the author(s) for this preprint version do not permit archiving in PMC. The full text is available from the preprint server.

